# Fuzzy classification of phantom parent groups in an animal model

**DOI:** 10.1186/1297-9686-41-42

**Published:** 2009-09-28

**Authors:** Freddy Fikse

**Affiliations:** 1 Department of Animal Breeding and Genetics, Swedish University of Agricultural Sciences, Box 7023, 75007 Uppsala, Sweden

## Abstract

**Background:**

Genetic evaluation models often include genetic groups to account for unequal genetic level of animals with unknown parentage. The definition of phantom parent groups usually includes a time component (e.g. years). Combining several time periods to ensure sufficiently large groups may create problems since all phantom parents in a group are considered contemporaries.

**Methods:**

To avoid the downside of such distinct classification, a fuzzy logic approach is suggested. A phantom parent can be assigned to several genetic groups, with proportions between zero and one that sum to one. Rules were presented for assigning coefficients to the inverse of the relationship matrix for fuzzy-classified genetic groups. This approach was illustrated with simulated data from ten generations of mass selection. Observations and pedigree records were randomly deleted. Phantom parent groups were defined on the basis of gender and generation number. In one scenario, uncertainty about generation of birth was simulated for some animals with unknown parents. In the distinct classification, one of the two possible generations of birth was randomly chosen to assign phantom parents to genetic groups for animals with simulated uncertainty, whereas the phantom parents were assigned to both possible genetic groups in the fuzzy classification.

**Results:**

The empirical prediction error variance (PEV) was somewhat lower for fuzzy-classified genetic groups. The ranking of animals with unknown parents was more correct and less variable across replicates in comparison with distinct genetic groups. In another scenario, each phantom parent was assigned to three groups, one pertaining to its gender, and two pertaining to the first and last generation, with proportion depending on the (true) generation of birth. Due to the lower number of groups, the empirical PEV of breeding values was smaller when genetic groups were fuzzy-classified.

**Conclusion:**

Fuzzy-classification provides the potential to describe the genetic level of unknown parents in a more parsimonious and structured manner, and thereby increases the precision of predicted breeding values.

## Background

Historically, genetic groups have been included in genetic evaluation models to account for selection not described by known genetic relationships. Introduction of animal models, which makes is possible to account for known relationships in the genetic evaluation, reduced the need for genetic groups [1,2]. However, in practice, genetic evaluations can by hampered by incomplete pedigrees, due to, for example, deficiencies in pedigree recording systems and importation of animals.

An animal whose parent(s) are unknown can be assigned so-called phantom parents. These phantom parents are assumed to be unrelated, non-inbred and to have a single descendant. Phantom parents themselves are not of interest, but are considered only to facilitate modelling and computations [3].

The strategy for assigning unknown parents to genetic groups should reflect the average genetic level of unknown parents [1]. Differences in the genetic level between sub-populations of unknown parents are a good reason to form genetic groups for each sub-population, thereby avoiding the assumption that base animals belong to a single population. Common factors considered in the definition of genetic groups for unknown parents are birth year of progeny, selection intensity (selection path) and origin [3-5].

Except for a few examples where genetic groups are clearly distinct, definitions of genetic groups are often based on arbitrary rules. An accurate modelling of the expected breeding values of unknown parents will lead to the creation of many groups, each one with only a few animals. However, the drawbacks of such a strategy are either confounding with other fixed effects in the model [6] or imprecise solutions for genetic group effects. Thus, definition of genetic groups should not be too precise to yield sufficiently large groups. Moreover, if incorrect information about a base animal's attributes (e.g., birth year and origin) is used to assign the unknown parents to a group, then genetic groups will not reflect the expected genetic merit of the unknown parents. These aspects of uncertainty and inaccuracy are usually not adequately handled in the allocation of unknown parents to genetic groups.

To improve the adjustment for seasonal effects in the genetic evaluation, Strandberg and Grandinson [7] have suggested to assign a cow's record partially to the herd-year-season of calving, and partially to the closest adjoining class. They have labelled the approach "fuzzy classification", after fuzzy logics, a methodology used in expert systems to handle inexact reasoning.

The aim of this study was to develop an algorithm for fuzzy classification of phantom parent groups in an animal model. This approach is illustrated with a simulation, where fuzzy-classified genetic groups are used to handle uncertainty about the time of birth. In addition, the possibility to model a linear time trend in the average genetic level of phantom parents with a small number of genetic groups will be illustrated.

## Methods

### Genetic model

The infinitesimal model assumes that an animal's breeding value is the sum of the parent average and a Mendelian sampling deviation that resembles the random process of sampling parental genotypes (e.g. [8]):

where *u*_*i *_and *N*_*i *_are the additive genetic value and Mendelian sampling deviation for animal *i*, respectively, and *s *and *d *are the sire and dam of animal *i*. In matrix representation:

where:

**u**_**b **_= vector of additive genetic effects for phantom parents;

**u **= vector of additive genetic effects for known animals;

[**P**_**b **_**P**] = matrix that relates parents to progeny; each row contains two non-zero elements (0.5) in the columns pertaining to the sire and dam;

**v **= vector with Mendelian sampling deviations.

Rearranging this yields the following result [6]:

That is, the additive genetic merit of all animals can be written as a linear function of the additive genetic effects of phantom parents and Mendelian sampling deviations.

### Genetic groups

Inclusion of genetic groups in the model for genetic evaluation can accommodate for non-zero base population means:

where:

**Q**_**b **_= incidence matrix relating phantom parents to their respective base population means;

**g **= vector with base population means.

In the approach by Robinson [5] and Quaas [6], the matrix **Q**_**b **_contains one non-zero element in each row, in the column corresponding to the genetic group to which the phantom parent belongs. For the fuzzy classification it is proposed that any row of matrix **Q**_**b **_has all elements equal to 0, except for one, two or more non-zero coefficients (elements defined by 0 ≤  ≤ 1) such that they add up to 1. For example, if the birth year of a base animal is estimated to be *yr*, the phantom parent of this animal can be allocated to genetic groups for birth year *yr-1*, *yr *and *yr+1 *with proportions 0.2, 0.6 and 0.2. This way it is possible to accommodate for uncertainty about the attributes of the base animal that are used for allocation of its phantom parents to genetic groups.

As a consequence of the non-zero base population means, the expectation of the vector with additive genetic effects becomes [6]:

where:

Matrix (**I - P**)^-1^**P**_**b **_relates the breeding values of animals to phantom parents. A single non-zero element in a row represents the expected fraction of the i^th ^animal's genes derived from the j^th ^phantom parent, and the rows of this matrix sum to one [6]. Post-multiplication of this matrix with the "fuzzy" **Q**_**b **_yields a matrix the rows of which sum to one, for both the distinct and fuzzy classification of genetic groups. Element *q*_*ij *_of **Q **is the expected fraction of the *i*^th ^animal's genes deriving from the *j*^th ^base population. Matrix **Q **can be computed recursively from a list of sires and dams. Element *q*_*ij *_= 0.5(S + D), where S (or D) is *q*_*sj *_(or *q*_*dj*_), or the proportion of phantom parent of animal *i *assigned to group *j*.

#### Example

**Figure 1:**
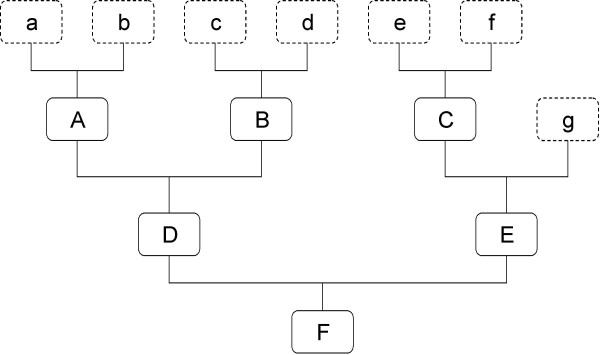
**Pedigree example**.

Consider the example pedigree in Figure 1. Upper case letters refer to identified animals, and lower case letters to their unknown base parents. The matrices **P**_**b**_, **P**, (relating parents to progeny) and (**I-P**)^-1^**P**_**b **_(relating animals to phantom parents) for this example pedigree are:

Note that matrix (**I - P**)^-1^**P**_**b **_represents a block of matrix **T **that would arise from the **TDT' **decomposition (where **D **is a diagonal matrix and **T **a lower triangular matrix; [9]) of the relationship matrix for unknown parents a-g and know animals A-F.

Suppose that phantom parents can be related to their (three) base groups according to the following matrix:

Here, phantom parents a and c came from one group, phantom parent b came from another group, and phantom parent e from a third group. Phantom parents d and f were partly assigned to the second group and the remainder to a third group, and phantom parent f was assigned to all groups, with proportions summing to one. Consequently,

Observe that the rows of matrix **Q **sum to one. In the original specification with distinct genetic groups, matrix **Q **would contain sums of powers of 0.5 as elements. In case of fuzzy- classified genetic groups, **Q **can essentially contain any value between 0 and 1, depending on the fractions in **Q**_**b**_.

### Mixed model equations

The transformed mixed model equations for an animal model with genetic groups derived by Quaas [6] are also applicable in the case of fuzzy classification of genetic groups, because the derivation is for any arbitrary matrix **Q**. The part on the left hand side is due to relationships:

where **A**^-1 ^is the additive genetic relationships between known animals and **Q**, as defined before, relates breeding values of known animals to genetic group effects [6].

Considering that **A**^-1 ^= (**I - P'**)**D**^-1^(**I - P**) [9] and drawing on the definition of **Q **in 2.2, Quaas [6] showed that A* for a model with genetic groups can be written as:

where  is the *i*^th ^row of **H**, *d*_*i *_is the *i*^th ^diagonal element of **D**, **H **= [**(I-P) **-**P**_*b*_**Q**_*b*_], and **D**^-1 ^= var(φ). For animals with known parents, each row of **H **contains three non-zero elements: a one and two negative halves. For base animals with phantom parents, each row of **H **usually contains three or more non-zero elements: a 1 and several negative values corresponding to the phantom parents' groups. These elements have a value of -0.5*p*_*ij*_, where *p*_*ij *_is the proportion of phantom parents of *i *assigned to group *j*. This structure leads directly to an algorithm for forming A* that is a simple extension of Henderson's **A**^-1 ^algorithm. Let *s*_*i *_(*d*_*k*_) be the number of groups the phantom sire (dam) of *i *is assigned to, or 1 if the sire (dam) of *i *is known; *m*_*j *_(*f*_*k*_) the equation number of the animal's sire (dam) or its group if the sire (dam) is unknown, and x = , then the following contributions need to be added: see table 1.

**Table 1:** Contributions to the mixed model equations in the case of fuzzy classification of genetic groups

Position	Contribution	
(i, i)	x	
(i, m_j_), (m_j_, i)	-0.5 p_ij _x	j = 1,..., s_i_
(i, f_k_), (f_k_, i)	-0.5 p_ik _x	k = 1,..., d_k_
(m_j_, m_j'_)	0.25 p_ij _p_ij' _x	j = 1,..., s_i_;
		j' = 1,..., s_*i*_
(f_k_, f_k'_)	0.25 p_ik _p_ik' _x	k = 1,..., d_k_;
		k' = 1,..., d_k_
(m_j_, f_k_), (f_k_, m_j_)	0.25 p_ik _p_ik' _x	j = 1,..., s_i_;
		k = 1,..., d_k_

The rules for creating A* resemble those for forming the inverse of an average numerator relationship matrix in case there are a finite number of potential parents (e.g., [10,11] and [12]).

#### Example: creating A*

For the example pedigree (Figure 1), the contributions for animals A-D to elements of A* are in Table 2. For animals A, D and F there are 9 contributions to A*, for animal B and C 16,

**Table 2:** Contributions to A* for animals A, B, C and D in the pedigree example

A	B	C	D
1.0 to A,A	1.0 to B,B	1.0 to C,C	2.0 to D,D
-0.5 to A,g_1_	-0.5 to B,g_1_	-0.15 to C,g_2_	-1.0 to D,A
-0.5 to g_1_,A	-0.5 to g_1_,B	-0.15 to g_2_,B	-1.0 to A,D
-0.5 to A,g_2_	-0.3 to B,g_2_	-0.35 to B,g_3_	-1.0 to D,B
-0.5 to g_2_,A	-0.3 to g_2_,B	-0.35 to g_3_,B	-1.0 to B,D
0.25 to g_1_,g_1_	-0.2 to B,g_3_	-0.5 to B,g_3_	0.5 to A,A
0.25 to g_1_,g_2_	-0.2 to g_3_,B	-0.5 to g_3_,B	0.5 to A,B
0.25 to g_2_,g_1_	0.25 to g_1_,g_1_	0.0225 to g_2_,g_2_	0.5 to B,B
0.25 to g_2_,g_2_	0.15 to g_1_,g_2_	0.0525 to g_2_,g_3_	0.5 to B,B
	0.15 to g_2_,g_1_	0.0525 to g_3_,g_2_	
	0.1 to g_1_,g_3_	0.075 to g_2_,g_3_	
	0.1 to g_3_,g_1_	0.075 to g_3_,g_2_	
	0.09 to g_2_,g_2_	0.1225 to g_3_,g_3_	
	0.06 to g_2_,g_3_	0.175 to g_3_,g_3_	
	0.06 to g_3_,g_2_	0.175 to g_3_,g_3_	
	0.04 to g_3_,g_3_	0.25 to g_3_,g_3_	

and for animal E 25. For animal C there are 16 contributions, but only 9 elements of A* are

affected because the phantom parents of animal C are (in part) assigned to the same group. Observe that the values added to A* can become very small if phantom parents are assigned to genetic groups with low proportions, for example 0.04 to element (g_3_, g_3_) for animal B.

The complete A* for this example is:

## Simulation studies

A population subject to mass selection was simulated for 10 non-overlapping generations, subsequent to a base population of unrelated and non-inbred animals (generation 0). Each generation, 50 males and 200 females were randomly mated. Each mating produced two offspring, one of each gender.

For each animal a phenotypic record was simulated as the sum of an overall mean, the animal's breeding value and a random residual. An animal's breeding value was generated as the sum of the parent average and a Mendelian sampling deviation that considered inbreeding of the sire and dam. Genetic and residual variances were both 10, yielding a heritability of 0.50.

For each replicate, a "real life", incomplete data set for genetic evaluation was created by randomly deleting data. When data were deleted, both phenotypic records and relationship were deleted. The probability of deletion decreased linearly with increasing generation number, and ranged between 0.30 (generation 10) and 0.70 (generation 0).

The model for genetic evaluation included an overall mean, random animal effect and genetic groups for phantom parents. The simulated variance components were used to predict breeding values. Solutions to the mixed model equations were obtained using the preconditioned gradient algorithm, which was assumed to be converged when the relative average difference between the right and left hand sides was smaller than 10^-10^.

Fuzzy classification was compared with distinct classification of genetic groups in two situations: 1) to handle the uncertainty about which group a phantom parent should be assigned to, and 2) to model the average genetic level of unknown parents with a small number of parameters.

### Fuzzy classification to handle uncertainty

For the genetic evaluation and forming of genetic groups, uncertainty about the generation of birth was simulated for 25% of the animals with at least one unknown parent. The uncertainty was such that unknown parents could belong to two possible generations: the true generation of birth and the generation prior to that, each with equal probability (0.50). Phantom parents were grouped based on gender and generation number, which resulted in 20 different genetic groups. In the distinct classification, for animals with simulated uncertainty, phantom parents were randomly assigned to just one genetic group, either for the true generation of birth or the generation prior to that, with equal probability. In the fuzzy classification, these phantom parents were assigned to two genetic groups, for both possible generations, with equal proportions (0.50).

The simulation was repeated 50 times. For each replicate the empirical mean and variance of prediction errors (true minus predicted breeding value) were computed within generation for animals with simulated uncertainty. In addition, the rankings on predicted breeding values for distinct and fuzzy classification were compared with the rankings on true breeding values. Animals were grouped in deciles based on the predicted and true breeding value, and the percentage of animals classified in the correct decile was determined for animals with and without simulated uncertainty and for animals with both parents known.

### Fuzzy classification for parsimonious modelling

Two strategies for assigning phantom parents to genetic groups were compared: distinct and fuzzy classification. In the distinct classification, phantom parents were grouped on the basis of gender and generation number, which resulted in 20 different genetic groups. The average number of animals per genetic group was 107 and group size ranged between 59 and 146. In the fuzzy classification there were four groups: two groups for the parent's gender (male, female) and two groups to describe the average genetic level of parents of generation 1 animals and one for parents of generation 10 animals. Phantom parents of animals in intermediate generations were assigned to both groups, with proportions depending on generation number. For example, a phantom sire of an animal born in generation two was assigned with 50% and 0% to the male and female parent genetic group and with 45% to the generation 1 genetic group and with 5% to the generation 10 genetic group, for a phantom dam of an animal born in generation three the proportions were 0%, 50, 40% and 10%, respectively, etc. This way, the sum of proportions was always equal to 1. The resulting **Q **matrix is not of full rank, meaning that only 3 degrees of freedom are used to model the genetic groups.

The simulation was repeated 50 times. For each replicate, the empirical mean and variance of prediction errors (true minus predicted breeding value) were computed within generations, separately for animals with 0, 1 or 2 unknown parents. The across-replicate standard deviation of estimates for each genetic group (20 and 3 in case of distinct and fuzzy classification, respectively) was also inspected as an indication of the SE of genetic group solutions. In addition, the across-replicate correlation between genetic group estimates was computed as an indication of sampling correlation of genetic group solutions.

## Results

### Fuzzy classification to handle uncertainty

The empirical prediction error variance was 1.6% lower (P < 0.05) for animals with simulated uncertainty when genetic groups were fuzzy-classified. For animals with unknown parent groups and for which the generation of birth was accurately known there was no difference in empirical prediction error variance between both grouping strategies. For animals with simulated uncertainty, the percentage of correctly ranked animals was higher (0.7 percent point; Table 3). More importantly, accounting for the uncertainty reduced the variation across replicates by more than 10% (Table 3).

**Table 3:** Percentage of animals correctly classified in deciles created on the basis of ranking on true breeding values.

*Group of animals*	*Distinct classification*		*Fuzzy classification*	
	
	Mean	SD	Mean	SD
Both parents known	39.7	1.4	39.6	1.4
Unknown parent(s) - simulated uncertainty	41.4	3.3	42.1^a^	2.9^a^
Unknown parent(s) - no uncertainty	41.1	3.3	41.2	3.1

^a ^Significantly different from distinct classification (P < 0.05)

The magnitude of the advantage of fuzzy classification is specific for this simulation design and will vary from case to case, whether it concerns other simulation designs or practical applications. The gains presented here represent a best case scenario, since some aspects of the pattern of uncertainty could be considered in the membership functions. In practical applications this will not be the case and there will be some noise even with fuzzy-classified groups.

### Fuzzy classification for parsimonious modelling

**Figure 2:**
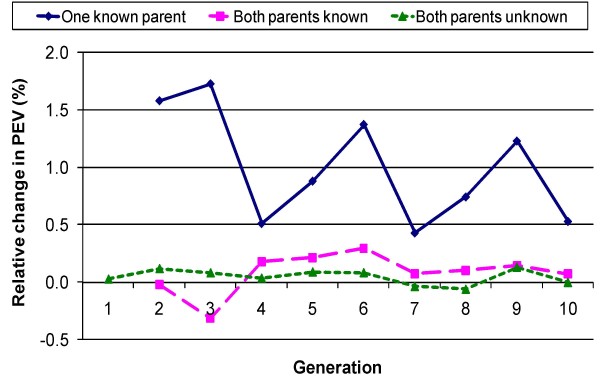
**Relative difference (distinct *vs*. fuzzy classification; %) in prediction error variance for animals having 0, 1 or 2 phantom parent(s), by generation number**.

The empirical prediction error variance was significantly (P < 0.05) lower when genetic groups were fuzzy-classified for animals with one unknown parent (Figure 2). The improvement ranged between 0.4% and 1.7%, depending on generation number. The SE of genetic group solutions was approximately 1.8 times lower for fuzzy-classified groups compared to distinct genetic groups. In case of fuzzy classification, much fewer groups (about six times less) were needed to describe the linear pattern of genetic trend of unknown parents, resulting in a higher precision of the genetic group solutions and explaining the lower prediction error variance of breeding values for this alternative.

The correlation between solutions for genetic groups for phantom sires and dams (for the same generation) was moderately negative (~-0.3) for the distinct classification of genetic groups, but slightly positive (~0.1) when groups were fuzzy-classified (results not shown). This result may explain why the prediction error variance differed between alternatives for animals with one phantom parent, but not for animals with two phantom parents. For an animal with two unknown parents, overestimation of the solution for the genetic group effect for one parent can be compensated by an underestimation of the solution for the genetic group effect for the other parent. When only one parent is unknown such compensation does not occur, and a more precise estimation of the genetic group effects, as in the fuzzy-classified alternative, is favourable.

## Discussion

The rules for building the numerator relationship matrix in the case of fuzzy-classified groups are similar to those for building an average numerator relationship for cases where parenthood is uncertain, but limited to a small number of possible parents (see rules in [10,11], and [12]). The conceptual difference between both procedures is that for the average relationship potential parents are known, whereas in the case of (fuzzy-classified) genetic groups parents are unknown.

Incorporation of genetic groups in the genetic evaluation can have a substantial effect on the estimated genetic trend and selection of which parents to breed the next generation of animals (e.g. [13]). Inclusion of genetic groups may sometimes lead to incorrect ranking of animals and suboptimal selection decisions [14]. Therefore, for practical applications it is important to evaluate the consequence of different grouping approaches (distinct, fuzzy) and definition of genetic groups (on the basis of birth year, selection path, origin, etc., or combinations thereof) before deciding which one to adopt.

Membership functions should follow the pattern of uncertainty and accurately describe the average genetic level of phantom parents as much as possible. However, consideration should also be given to the precision and the estimability of genetic group effects when determining the proportions with which phantom parents are assigned to genetic groups. For example, very small values are added to the diagonal elements of the mixed model equations for a genetic group to which phantom parents are assigned with small proportions, resulting in a high standard error for that genetic group. Also, to avoid confounding among genetic groups it is necessary to have several combinations of different genetic groups and for the same combination of genetic groups to use several different sets of membership proportions. Therefore, in the evaluation of fuzzy-classified genetic groups it is important to carefully examine the precision of genetic group estimates and possible confounding of genetic groups.

A linear trend in the average genetic level of phantom parents was modelled with a low number of parameters (groups) by fuzzy-classification in the simulation. More complex modelling of the average genetic level of phantom parents is possible by means of the fuzzy-classification approach. For example, other factors, like origin and selection path, could be incorporated in a similar way.

## Competing interests

The author declares that they have no competing interests.

## Authors' contributions

WFF conceived and designed the study, programmed and carried out the computer simulations, analyzed and interpreted the results, and wrote the manuscript.
